# The cost effectiveness of vaginal versus abdominal repair of vesicovaginal fistulae

**DOI:** 10.1007/s00192-019-04015-7

**Published:** 2019-07-18

**Authors:** Ross Warner, Alice Beardmore-Gray, Mahreen Pakzad, Rizwan Hamid, Jeremy Ockrim, Tamsin Greenwell

**Affiliations:** Department of Urology, University College London Hospital at Westmoreland Street, 16–18 Westmoreland Street, London, W1G 9PH UK

**Keywords:** Cost-effectiveness, Length of stay, Martius fat pad, Omental flap, Reconstructive urology, Vesicovaginal fistula

## Abstract

**Introduction and hypothesis:**

The objective was to assess the comparative provider costs of vaginal and open abdominal repair of vesicovaginal fistula (VVF) and to determine the most cost-effective means of managing VVF.

**Methods:**

A prospectively acquired database of all women undergoing VVF repair by a single surgeon between 2007 and 2015 was retrospectively reviewed to determine operating time, perioperative complications, inpatient stay and 30-day readmissions. The success and cost of the VVF repair were identified. Statistical analysis was by unpaired* t* test, Chi-squared test and Mann–Whitney* U* test.

**Results:**

Forty-seven consecutive women of mean age 51 years (range 21–88) undergoing a first attempt at VVF repair at our institution were included; 32(68%) had vaginal repair with Martius fat pad interposition and 15 (32%) had open abdominal repair with omental interposition. There were no perioperative complications or 30-day readmissions in either group. Mean operative time was longer for open abdominal (223.4 min) than vaginal repair (196.9 min). Median inpatient stay was longer for an open abdominal (8 days) than for a vaginal approach (4 days). Successful anatomical closure was achieved in 91% of vaginal and 86% of open abdominal repairs at first attempt, and in 100% after second repair, where required. Mean/median costs for an abdominal repair were significantly higher, at £4,608.69/£4,169.20 than for vaginal repair at £3,381.50/£3,009.24 (*P*<0.05).

**Conclusions:**

Vesicovaginal fistulae were successfully repaired in 89% of cases at first attempt. The success rate did not differ between approaches. Vaginal repair is significantly more cost-effective than abdominal repair owing to the shorter operative time and length of stay.

## Introduction

Vesicovaginal fistulae (VVF) cause significant morbidity. For patients, the impact on their quality of life can be devastating and for healthcare services, the costs of treatment and repair is high. In the UK and developed world, VVF are most commonly caused by iatrogenic injury following obstetric, gynaecological or urological surgery [1]. Other recognized causes include local invasion as a result of pelvic malignancy and radiotherapy. There are multiple recognized surgical approaches to repairing VVF, as summarized in previous reviews [2, 3]. An important surgical consideration is whether to use a transvaginal or a transabdominal approach. It has previously been established that vaginal approaches lead to a shorter hospital stay and quicker recovery times [4, 5]. Current evidence suggests that where both routes are an appropriate surgical option, there might be similar success (radiologically confirmed closure) [6], quality of life and sexual function [7]. In this paper, we have analysed a single-surgeon series of VVF repairs to consider the cost implications for healthcare providers of an open transabdominal versus a transvaginal approach.

## Materials and methods

### Data collection

A retrospective analysis of a prospectively collected database of all urinary tract fistula repairs at a single institution carried out by a single surgeon was performed for all cases of VVF repair between January 2007 and December 2015. Additional data were collected from patient case notes, electronic records and our hospital finance and procurement departments.

### Inclusion/exclusion criteria

Repairs of fistulae involving other urogenital structures, including urethrovaginal and ureterovaginal fistulae were excluded, as were abdominal VVF repair operations at which additional procedures (such as ureteric reimplantation) were performed and recurrent fistulae with previous repairs at our or other institutions. For the patients included, there were no absolute indications for repair by one technique or the other.

### Surgical technique

At our institution vaginal repair is performed with a modified Martius labial fat pad flap interposition (mMlfpf) and open abdominal repair with omental flap interposition. Choice of approach was based on surgeon preference with an evolution from the initial use of a predominantly open abdominal technique to a predominantly vaginal approach over time. All patients had preliminary cystoscopy and placement of a Pollock open-ended ureteric stent through their fistula with placement of bilateral or unilateral Pollock catheters intra-ureterically if the fistula was close to the ureteric orifices.

### Outcomes

Primary outcomes were the mean cost of repair per patient for each surgical approach and mean total cost per successful repair. Secondary outcomes included duration of operating time, peri-operative complications, length of hospital stay, 30-day readmission rate, success or not of the repair and fistula complexity. A 1-day length of stay was considered as being in hospital overnight into the next day following surgery. A 30-day readmission rate was used to identify any complications outside the immediate post-operative period that would impact on hospital costs. Success was defined by post-operative imaging (cystogram) confirming closure of the fistula and resolution of continuous incontinence confirmed by patients on follow-up outpatient assessment, at 4 and 12 months. Fistula complexity was measured according to the Goh classification, a three variable measure that looks at fistula size, distance from the external urethral orifice and scarring [8].

### Cost calculations

Our institution is a National Health Service (NHS) provider in the UK. There are no costs to a patient undergoing VVF repair. Cost evaluations have therefore been performed from a healthcare provider perspective.

Costs were identified from the Service-Line Reporting (SLR) tool for our institution. SLR is a measure of the cost/profitability of a particular service set out by the independent regulator of NHS trusts in the UK—NHS Improvement (formerly Monitor) [9]. In this instance, the service cost includes the pre-operative admission, theatre session, post-operative theatre recovery, inpatient bed days and all resources necessary to provide this, including staff and standard equipment. Figures correct at the time of the final included operation (December 2015) were used to calculate the cost of all procedures, to control for inflation.

### Statistical analysis

Statistical analysis was by unpaired* t* test, Chi-squared test and Mann–Whitney* U* test.

### Ethical approval

Ethics/Institutional Review Board approval was not required, as our institution considers this type of review to be a service evaluation and not a study.

## Results

**Fig. 1:**
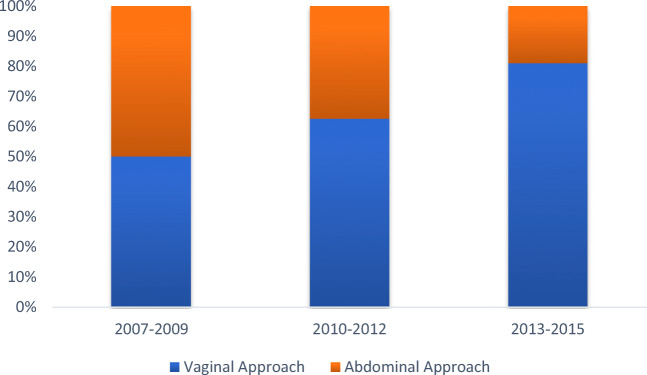
Change in proportion of operations carried out by a vaginal or abdominal approach over time

Forty-seven consecutive women undergoing a first attempt at VVF repair at our institution, by the same surgeon, between 2007 and 2015, were included. The mean age was 51 years (range 21–88). Over this time, 32 women (68%) had a vaginal repair with modified Martius labial fat pad flap interposition and 15 (32%) had open abdominal repair with omental flap interposition. The distribution of operations over time can be seen in Fig. 1.

A greater proportion of patients in the vaginal repair group had a malignant aetiology for their fistula compared with the open abdominal group, although this difference was not statistically significant (Table 1). There was also no significant difference between the two treatment groups in terms of fistula size, *p*=0.65 (Table 2), or fistula complexity (Table 3).

**Table 1:** Demographics of the vaginal and abdominal vesicovaginal fistula (VVF) repair technique groups

	Vaginal repair (*n*=32)	Abdominal repair (*n*=15)
Median age (years) (range)	48 (21–88)	49 (32–80)
Percentage of malignant fistulae	31	13
Aetiology of fistula (% of all fistulae for each approach)		
Gynaecological surgery	22 (68.8)	11 (73.3)
Obstetric surgery	3 (9.4)	2 (13.3)
Urological surgery	5 (15.6)	2 (13.3)
Radiotherapy	1 (3.1)	–
Foreign body	1 (3.1)	–

**Table 2:** Comparison of fistula size between the vaginal and abdominal repair groups. *p*>0.05

	Vaginal repair	Abdominal repair
Mean fistula size (cm)	1.47	1.28
Median fistula size (cm)	1	1
Range fistula size (cm)	0.5–4	0.5–2

**Table 3:** Comparison of fistula complexity between the vaginal and abdominal repair groups

Goh classification [8]	Vaginal repair	Abdominal repair
1aI	13 (40.6)	5 (33.3)
1aII	1 (3.12)	3 (20)
1aIII	3 (9.38)	1 (6.67)
1bI	6 (18.75)	2 (13.33)
1bII	2 (6.25)	2 (13.3)
1bIII	0	0
1cI	0	0
1cII	0	0
1cIII	1 (3.12)	0
2aI	3 (9.38)	1 (6.67)
2aII	0	1 (6.67)
2aIII	0	0
2bI	1 (3.12)	0
2bII	0	0
2bIII	0	0
2cI	1 (3.12)	0
2cII	0	0
2cIII	0	0
3aI	0	0
3aII	0	0
3aIII	0	0
3bI	0	0
3bII	0	0
3bIII	0	0
3cI	1 (3.12)	0
3cII	0	0
3cIII	0	0

The costs of the key factors that are well established to vary between the two operative techniques—operative time and length of stay—were identified from our institution’s SLR (Table 4).

**Table 4:** Key variable costs of patient pathway for VVF repair

Item	Cost
Operating theatre	£625.11 per hour^a^
Inpatient bed	£250.92 per day

Costs at our institution as of November 2015

^a^Includes cost of time in surgical admissions and post-operative recovery areas

No significant difference was identified in the intra-operative equipment costs, such as the use of ureteric stents, between the two procedures. No peri-operative complications were seen in our series meaning that other additional costs, such as packed red cell transfusions, management of infection and return to theatre, were not required. There were no 30-day readmissions.

Post-operative follow-up is similar at our institution for both techniques. Patients in both groups underwent a single cystogram 3 weeks following repair. There were no differences in the number of outpatient appointments required between the two groups.

There was a small increase in the mean operative time from a vaginal to an open abdominal approach, but this was not significant (Table 5). In contrast, there was a significant difference (*p*<0.05) in the inpatient length of stay, with patients undergoing a transabdominal approach staying approximately 4 days longer (Table 5).

**Table 5:** Comparison of duration of operation and inpatient stay between vaginal and abdominal VVF repair

	Vaginal repair	Abdominal repair
Mean operative time, min (range)	196.89 (85–351)	223.4 (88–380)
Median operative time (mins)	192.5	207.5
Mean inpatient stay, days (range)	5.3* (1–38)^a^	9.1* (4–20)
Median inpatient stay, days)	4**	8**

*p<0.05

**p<0.05

^a^Outlier of 38 days due to a single elderly patient with significant cardiac history

**Fig. 2:**
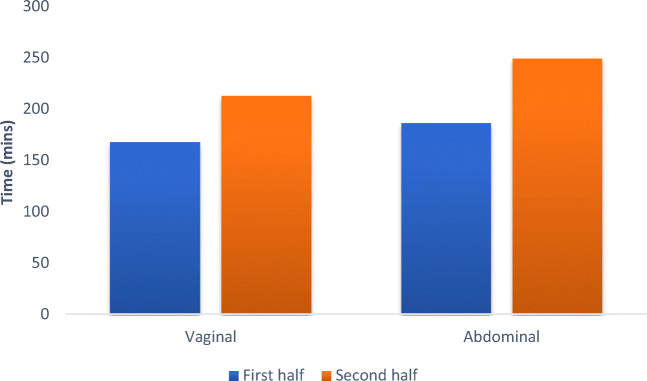
Comparison of** a** the median operative time and** b** the median length of stay between the first 50% and second 50% of operations performed via an abdominal and vaginal approach respectively

Assessment of the impact of the learning curve on the costs of each technique was made by comparing the operative time and length of inpatient stay of the first 50% of cases for each approach with the second 50%. Although a trend of increasing operative and admission time was identified towards later cases, this was not significant (Fig. 2).

The fistula was repaired successfully at first attempt in 89% of all cases. This corresponds to an 86% first time closure rate in the open abdominal group and a 91% first time closure rate in the vaginal repair group. There was no significant difference between the two groups in terms of success of the repair. In all vaginal cases, the fistula was successfully reached and repaired with a mMlfpf with no need for conversion to an open abdominal approach. Factors associated with failure included radiotherapy as aetiology, larger fistula and older age [10].

Variability in the costs between the two techniques was calculated using the data in Tables 4 and 5. This demonstrated a significant difference (*p*<0.05) in the mean/median total cost and in the mean/median total cost per successful repair between the two approaches (Table 6). In our series, the median cost per patient was £1,159.96 greater for those undergoing an open transabdominal VVF repair compared with a transvaginal approach.

**Table 6:** Cost comparison of vaginal vs abdominal VVF repair

	Vaginal repair cost	Abdominal repair cost
Operative time, mean (median)	£2,051.62 (£2,005.56)	£2,327.83 (£2,161.84)
Inpatient stay, mean (median)	£1,329.88 (£1,003.68)^a^	£2,280.86 (£2,007.36)^a^
Total cost, mean (median)	£3,381.50 (£3,009.24)^b^	£4,608.69 (£4,169.20)^b^
Cost per successful repair, mean (median)	£3,715.93 (£3,306.86)^c, d^	£5,358.94 (£4,847.91)^c, e^

*p*<0.05 for corresponding pairs of letters ^a^, ^b^ and ^c^

^d^91% success rate

^e^86% success rate

## Discussion

Vesicovaginal fistulae can be closed at first attempt in 91% of women undergoing vaginal and 86% of women undergoing an open abdominal repair at our institute. There is a longer operative time (although not statistically significant) and a highly significantly longer inpatient stay for open abdominal repair. Total costs per successful repair are significantly lower for vaginal repair of VVF.

### Surgical approach

Open transabdominal and transvaginal approaches to VVF repair have long been established. The decision about which surgical approach to use for VVF repair is often based upon surgeon experience and preference [11]. In a bid to provide assistance in making this decision, our series demonstrates an evolution in practice for a single surgeon from a predominantly abdominal approach to a predominantly vaginal approach. This progression was made in a bid to reduce the exposure of patients to the greater morbidity seen with an abdominal approach, as identified by previous studies [12]. Patients were not randomly allocated to one approach or the other, meaning that there remains a risk of case selection bias. However, as experience with the transvaginal technique improved over time, more complex fistulae were deemed accessible vaginally. This is supported by the fact that no significant difference in the complexity of fistulae, as measured by the Goh classification, between the two groups and that longer operating times were seen in the second 50% of all transvaginal repairs compared with the first 50%. Of note, we accept that the longer operating times seen in the second 50% of procedures carried out could also have been influenced by other factors, including the increased involvement of trainees, a change in our operating theatre computer system part way through our data collection period (resulting in more accurate time keeping), or simply chance.

Given the similar success and complication rates demonstrated here across the two approaches, and the cost benefit of a vaginal repair technique, the adoption of an open abdominal technique should be based on patient/fistula anatomical considerations alone. Key factors previously cited as necessitating an abdominal repair include: a small-capacity, fixed, inaccessible vagina; close proximity of the fistula to the ureter, making ureteric reimplantation a possibility; a high vaginally inaccessible position of the fistula; and the presence of other pelvic injuries necessitating an abdominal approach, such as ureteric obstruction [11]. As our experience with vaginal repair has evolved, we have determined that all of the above “absolute” indications (other than the presence of other pelvic injuries, such as associated colovesical fistula or ureteric obstruction, which necessitate abdominal repair) are actually relative and can be overcome.

### Success rate

In our series, the rate of success between the two approaches, and therefore the requirement for further surgery when there was a treatment failure were similar. This meant that the mean cost per successful repair remained in favour of a transvaginal approach. If further operative interventions are required following failure of repair, the cost per successful repair will clearly increase. For a similar success rate between approaches, the reduced post–operative inpatient length of stay suggests that a transvaginal approach to VVF repair might be more cost effective than a transabdominal technique.

Of note, calculation of the societal cost of a failed repair is beyond the scope of this article and therefore the true cost per successful repair is unknown. This is an important limitation of this evaluation. It is not clear if the cost to society, and a national healthcare system such as that in the UK, would be different for each of the techniques. However, the increased length of stay and morbidity associated with an open abdominal approach as reported elsewhere would suggest that the societal cost of a successful abdominal repair might be likely to be greater than that of a vaginal repair [12].

The success rate demonstrated in our data for transvaginal repair (91%) is similar to those of previously published series. Previous reported figures range from 82 to 98% [1, 5, 6, 13, 14]. This variation is likely a combination of differing techniques (for example, the use of and type of tissue used for interposition), patient selection—with more complex fistulae historically reserved for an abdominal approach, fistula aetiology, alternative definitions of “success” between studies and a wide variation in sample sizes (from 11 to 207 patients).

### Operative time

The mean operating times of the two approaches are similar in our data, although slightly longer for an open abdominal repair (197 [vaginal] vs 223 [abdominal] min). The longer operating times within the range were related to access difficulty via both routes due to patient habitus ±previous surgery-/radiotherapy-induced adhesions. There are minimal data in the published literature with which to compare this, however, where reported, a transvaginal approach has been shown to be significantly quicker. Kapoor et al. described a mean time of 98 mins for vaginal repair and 167 mins for open abdominal repair [5]. The difference here is likely a result of careful selection of “simple” fistulae by Kapoor et al. for a vaginal approach only. Compared with these previously published data, it is likely that our timings in the two techniques are similar because complex fistulae were repaired transvaginally.

Furthermore, the longer operating time seen for both of our approaches compared with that of Kapoor et al. is likely the consequence of differing definitions of “operative time,” in other words, at what point the timer is started and stopped. In our institute, the surgical start time is taken from commencement of positioning for preliminary cystoscopy and surgical end time is following application of the dressing and removal of drapes. Thus, this includes cystoscopy, passage of a stent across the fistula, and often insertion of ureteric stents before starting the fistula repair, all adding significant time to the procedure.

### Length of stay

Median length of hospital stay for a transvaginal repair (4 days) is half that of an open abdominal operation (8 days) in our series. It is widely accepted that this trend would be expected; however, there are again minimal data in the literature to support this. In the English NHS, the mean length of stay for all VVF repairs (vaginal and abdominal) between 2012 and 2017 has been 7–7.7 days [15]. Kapoor et al. describe a mean inpatient stay of 7 days for vaginal repair and 10 days for abdominal repair [5]. We demonstrate a clear reduction in length of stay when a vaginal approach is used. Although not specifically collated within our database, the reduced post-operative pain, earlier mobilization, ability to remain extra-peritoneal and avoid opening of the bladder, and recovery of bowel function, are likely to contribute to this finding. It is possible that healthcare systems with excellent out of hospital/community care facilities and easy access to medical review can attain shorter inpatient admission durations.

### Limitations

A key limitation of this study is the difficulty in generalizing our outcomes to other cohorts. Although the use of single-surgeon data ensures continuity in operative technique and therefore more valid comparison between approaches, inter-operator variability could significantly alter surgical time and subsequently cost. Furthermore, since our institution is a national healthcare organization, we have only considered the cost to the provider. When payer costs are factored in, in a private healthcare environment, this may alter the cost of each approach.

Although these data represent one of the larger published single-surgeon open VVF repair series, the numbers included remain small. Consequently, a lack of statistical significance where differences have been identified may be due to underpowering of the analyses, for example, differences in fistula aetiology between the two groups.

A final limitation is the lack of a cost comparison with newer surgical techniques, namely laparoscopic and robotic-assisted VVF repair. To provide consistency in surgical technique and a direct comparison between approaches, a single-surgeon series has been used for this study. Our unit does not routinely perform laparoscopic surgery for VVF repair. Over the last two decades a number of case reports and series have been published demonstrating good success rates of laparoscopic/robotic VVF repairs. These have been summarized in a relatively recent systematic review of 44 studies [16]. Owing to the significant heterogeneity of these reports, there is significant variation in outcomes with mean length of stay ranging from 1 to 8 days, operative time from 70 to 347 mins and success rate from 80% to 100%. For the most part, a laparoscopic or robotic-assisted approach is considered an alternative to open abdominal repair and has therefore not been compared directly with a vaginal approach to date. A cost comparison would be difficult owing to the large variation in intra-operative equipment costs between a laparoscopic/robotic and a vaginal approach, which was not encountered in our study.

## Conclusions

Our review suggests that with no significant difference in the success rates, a vaginal approach to VVF repair might be more cost-effective than an open abdominal repair. Patients spend significantly less time in hospital following vaginal repair and as a consequence the cost per repair is significantly lower. If a vaginal approach is possible, this should be the operation of choice for a first-attempt VVF repair.
